# Association of Plastic Surgeons of India Postgraduate Medical Education (APSI-PGME) Course: How Far Have We Reached?

**DOI:** 10.1055/s-0045-1804532

**Published:** 2025-02-24

**Authors:** Veena Kumari Singh, Ankur Bhatnagar, Ravi Mahajan, Vijay Kumar, Nitin Mokal, Hari Venkatramani

**Affiliations:** 1 Department of Burns and Plastic Surgery, All India Institute of Medical Sciences, Patna, Bihar, India; 2 Department of Plastic and Reconstructive Surgery, Sanjay Gandhi Post Graduate Institute of Medical Sciences, Lucknow, Uttar Pradesh, India; 3 Department of Plastic and Reconstructive Surgery, Amandeep Hospitals, Amritsar, Punjab, India; 4 Department of Plastic and Reconstructive Surgery, King George Medical University, Lucknow, Uttar Pradesh, India; 5 Department of Plastic and Reconstructive Surgery, Sir H. N. Reliance Foundation Hospital and Research Centre, Mumbai, Maharashtra, India; 6 Department of Plastic and Reconstructive Surgery, Ganga Hospital, Coimbatore, India

**Keywords:** plastic surgeons, India, postgraduate, course, APSI

## Abstract

**Background:**

The Association of Plastic Surgeons of India (APSI) felt the need to create an opportunity for the residents to participate in a mock viva voce patterned course on the national platform in front of examiners from the various centers and instill a feeling of confidence and healthy competition among them. Apart from the responsibilities of teaching departments running the formal plastic surgery training programs, the association also took upon itself to further strengthen the academics, especially of residents working at centers that lack regular teaching classes, and started the postgraduate medical education course in October 2022.

**Materials and Methods:**

The course module was divided into nine sections: one long case, five short cases, instrument, radiology, operative. It was conducted over 2.5 hours for two consecutive evenings on an online platform (apsi.vidocto.com). The flow comprised presentation by the resident with two faculty examiners for asking questions and two course mentors for observation followed by feedback to the resident on his/her performance. Course feedback was collected in a Google form.

**Results:**

A total of eight courses have been conducted to date. The online synchronous viewership varied from 83 to 172 (mean, 115.94 ± 34.01), out of which 62 to 67% (mean, 74.18 ± 4.06) were residents and 33 to 38% (mean, 40.28 ± 3.93) were faculty. These courses were also archived for later access. The feedback showed that 96.6% participants agreed that the objective to enhance knowledge was achieved and 100% agreed that such a program will benefit in gaining confidence. Thirty-one percent students rated their learning experience after the course as 10 on a scale of 1 to 10. The majority of the faculty and residents preferred the online mode.

**Conclusion:**

Being a maiden initiative of its kind, the APSI-PGME is a competency-driven course that has significantly enhanced the residents' confidence as a result of gain in knowledge and understanding.

## Introduction

Traditionally, plastic surgery training methodologies focused on subjective evaluations and summative examinations in a time-bound manner, often at the expense of procedural skill training. However, adult learning theory is rooted in andragogy, which is learner oriented, in contrast to teacher-oriented learning in pedagogy. In 2013, in the United States, the Accreditation Council for Graduate Medical Education (ACGME) created the “Next Accreditation System,” which made the residents reach their educational milestones based on certain core competencies throughout the entire duration of their training. 1 Since the traditional time-based model requires a predetermined length of training irrespective of learning style, pace, or activity, a competency-based model is appealing because it refocuses education on deliberate and relevant skill acquisition and retention. Hence, the need of the hour is the slow transition of plastic surgery training to competency-based medical education, thereby permitting residents to learn at their own pace to master each competency.

In India, competency-based medical education in the plastic surgery curriculum designed by the National Medical Commission (NMC) in 2022 is focused on the student to become a lifelong learner. 2 Lifelong learning is a key component of competency-based medical education (CBME), which has demonstrated a paradigm shift not only in the aspect of innovative teaching–learning methodologies but also from the traditional classroom to a combination including virtual classroom. 3 The incorporation of online learning creates a community of instructors and students who are responsible for supporting and interacting with each other, similar to the traditional setting. 4 To foster the merits of the current curriculum for plastic surgery residents that is competency based rather than time bound, the Association of Plastic Surgeons of India (APSI) designed a web-based continuous teaching course around the year including the components of self-directed learning such as critical thinking (problem-solving) skills, research, effective time management, and good communication skills. The course incorporated the concept of formative assessment (assessment for learning) mentioned in CBME with the primary purpose of reinforcing student learning and providing feedback on performance and remediation.

## Conceptualization: Why the Need?

The first author is a regular faculty for plastic surgery cases in the various mock examinations being conducted over many years by the Association of Surgeons of India (ASI), in both the physical and virtual modes. There was no such course on the pattern of mock examinations being run by the APSI, although the APSI-accredited courses were conducted at regular intervals for resident teaching. The first author also happens to be an external examiner for plastic surgery courses at different medical colleges and observed the lacunae in the presentation of cases or other sections like instruments, radiology, operative viva, and so in the confidence of the residents who did not have exposure to the regular classroom teaching during their training period. The need for starting a structured teaching program in the form of an examination viva was strongly felt and a proposal was sent to the executive council of APSI, which was unanimously approved by all members. The inspiration for the ASPI-PGME courses was derived from the ASI Regional Refresher Course (RRC), which is held from July to September every year in all the zones of the country with immense benefit to all surgery trainees and faculty. 5 Apart from the responsibilities of teaching departments running the formal training programs, the association took it upon itself to further strengthen the academics of its postgraduate students. The core concept was to create an opportunity for the residents to appear for a mock viva voce on a national platform in front of examiners from the various centers and instill a feeling of confidence and healthy competition among the residents.

## Learning Objectives

In India, classroom teachings in the form of case presentations, seminars, journal clubs, and operative chats are regular features at most of the departments running the plastic surgery courses, and a similar pattern is followed at the time of exit examinations with further inclusion of spotters, specimens, radiology, and osteology. So, the learning objectives of the course were to encourage the residents to recall the relevant anatomy and details of the disease process, describe the clinical presentation of the patient, demonstrate the examination of the relevant part, describe the tests that are commonly done in clinical practice relevant to the diagnosis, understand the planning of particular case management, understand the type of surgical procedures in the context of the patient presentation, demonstrate the decision-making skills, and write a summary of the case.

## Materials and Methods

### Designing of the Module

The first and the second authors were assigned the responsibility of designing the course module that was drafted in a month and validated by the other office bearers of the APSI. The module mentioned the need for such a course, its objectives, the flow of the course, guidelines for the residents, faculty examiners and mentors, expected benefits of the course, financial implications, a list of the cases to be covered, the centers, and their annual intake of residents, and the registration form for the faculty and residents ( Supplementary Material 1 , available in the online version).

### Flow of the Course

**Fig. 1:**
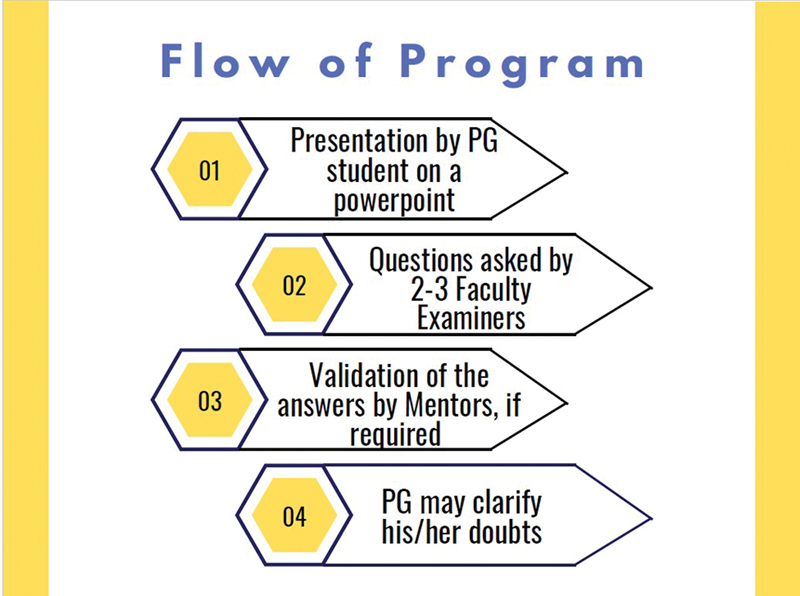
Step-by-step flow of the sessions during the course.

Each course was spread over 2.5 hours for two consecutive evenings (total, 5 hours) with nine sessions: one long case, five short cases, one instrument set, radiology including X-rays, orthopantomogram, computed tomography scans, and operative viva. Each session was conducted in four steps ( Fig. 1 ). The residents presented the cases in all sections using PowerPoint in a format similar to that used in classes or during exit examinations. A panel of two mock examiners then cross-questioned on the various aspects of the case. In case of incorrect answers by the student, the examiners further guided them toward the correct concepts and decision-making. Two mentors or observers, who were the senior examiners and past presidents of APSI, were given the responsibility of validating the answers in case there was no consensus among the examiners. The presence of a few senior examiners was an integral part as they standardized the answers in areas of doubt so that students from any center would come to know the correct answers relevant to the cases rather than getting confused. A feedback was provided to the students on one-to-one basis throughout the course so that they become aware of their performance and take the remedial measures to improve. The examiners and mentors pointed out the areas well presented by the participants and areas in which they lack knowledge or concept. They also summarized the key points at the end of every session, following which students were encouraged to clarify their queries, if any.

### Expected Outcomes

The proposed expected benefits were twofold. First, the presenting residents will have a boost for preparing well for their practical or viva voce with better self-directed learning skills, helping them in providing validated answers from the teachers and bringing uniformity in knowledge, gap, and practices of all residents irrespective of their training center, which will enhance their confidence. Second, the teachers will get the opportunity to teach other students apart from their institution, thus developing a feeling of satisfaction on seeing the students becoming more empowered and pursuing their passion of being a teacher.

## Actual Implementation

A Google Form was circulated every year to collect data regarding the resource faculty for the courses in that year. It included details like designation, APSI membership, whether they were a regular examiner, interest in being the faculty examiner for online courses, audiovisual arrangements for the web presentations, and contact details. The final list of faculties for a course was prepared after a one-to-one telephonic discussion regarding their availability, readiness, and the type of cases at their centers. The presenting resident was taken from the same college so that case selection could be finalized under the faculty's guidance. However, the same faculty used to be an examiner along with one more faculty in some other session. An announcement brochure for dates, scientific details, and registration was created by the APSI web partner for these courses. Free access was given to all APSI life and associate members, but minimal registration charge was kept for nonmembers. These courses were conducted online over 2.5 hours for 2 consecutive days in the evenings, a total of 5 hours per course. All faculty examiners, mentors, presenting residents, and organizers were logged in on the zoom platform and the sessions were livestreamed on https://apsi.vidocto.com/ for the viewers. After the course, all the sessions were archived on the same Web site for easy accessibility and ready reference for the APSI members. At the end of each course, a feedback on course module was collected and analyzed by the course directors for improvement. The data collected as responses to the questions were analyzed using descriptive statistics and percentage values. Thematic analysis was done for the qualitative data.

## Results

### First Course on October 13 to 14, 2022

APSI conducted the first PGME course from October 13 to 14, 2022 with technical support from its web partner. It was stretched over 2.5 hours for 2 days (a total of 5 hours) in the web-based mode. A total of 9 residents, 18 faculty members, and 2 mentors participated in the first course. On the first day, there was a long case discussion for 1 hour and three short cases for 30 minutes each. On the second day, two more short cases, instruments, radiology, and operative session, 30 minutes each, were held. The presenting residents, faculty examiners, mentors, and course directors were logged in via the zoom platform, and both days' sessions were livestreamed on the Association of Plastic Surgeons of India Postgraduate Medical Education (APSI-PGME) page separately created for this purpose. The success of the first course led to a decision to conduct at least four courses per year taking care of the schedule of plastic surgery exit examinations. It was also decided to give complimentary access to all APSI members and keep a nominal fee for the non-APSI members. To date, eight such courses have been conducted from October 2022 to June 2024 ( Supplementary Material 2 , available in the online version).

### Impact Measurement

After the completion of each course, a feedback on course module was collected from the participants in Google Form with a total of 11 items in three sections ( Supplementary Material 3 , available in the online version). The first section was related to details of the faculty and residents (their role, year of residency for students, whether they were an APSI member). The second section had four Likert scale questions in the context of time allotment for each section, whether the objective of the program to enhance knowledge of participants was achieved, the benefit of the students in gaining confidence, and rating of the course standard on a scale of 1 to 10. The third section had two open-ended and two close-ended questions: what else could have been included to enhance the learning, three things they did not like and could have been done differently, omitted, or modified, the preferred mode of program conduct, and the frequency of courses if conducted on the web-based mode.

In any course, the online viewership varied from 83 to 172 (mean, 115.94 ± 34.01), out of which 62 to 67% (mean, 74.18 ± 4.06) were residents and 33 to 38% (mean, 40.28 ± 3.93) were faculty members. Among the residents, 50% were final-year residents, 41.6% were second-year residents, and the rest were first-year residents. Among the viewers, 63.3% were APSI members, 26.7% were non-APSI members, and the remaining 10% applied for the APSI membership. This was a synchronous viewership that was taken into account for impact measurement. The courses were archived on the same platform so that they could be accessed later by others and those who want to refer again.

**Fig. 2:**
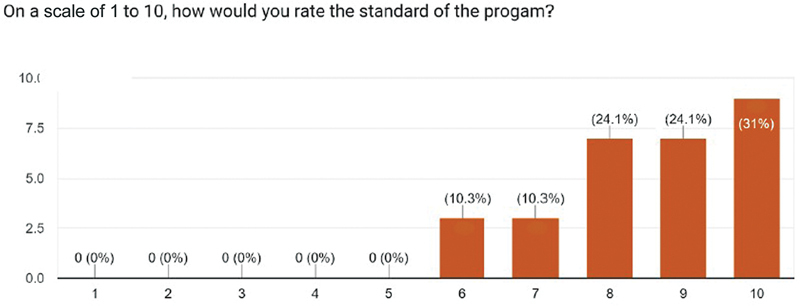
Rating of the course by participants.

For the appropriateness of the time allotted for each section, 89.7% marked it just right. In all, 55.2% strongly agreed and 41.4% agreed that the objective of the program to enhance the knowledge of participants has been achieved. Sixty-nine percent strongly agreed and 31% agreed that such a program will benefit the students in gaining confidence ( Table 1 ). The participants also rated their learning experience after the course on a scale of 1 to 10 in which the highest rating of 10 was given by the maximum number of students (31%), whereas a rating of 9 and 8 were given by 24.1% each, and the lowest ratings of 7 and 6 were given by 10.3% participants ( Fig. 2 ).

**Table 1:** Evaluation of responses to feedback questions using the Likert scale

	Likert's scale: 1–5	% of total	*p* -Value
The time allotted for each session was	Too fast	0	<0.001
	Fast	6.9	
	Just right	89.7	
	Slow	3.4	
	Too slow	0	
The objective of the program to enhance the knowledge of participants was achieved	Strongly agree	55.2	<0.001
	Agree	41.4	
	Neutral	3.4	
	Disagree	0	
	Strongly disagree	0	
Such a program will benefit the students in gaining confidence	Strongly agree	69	<0.001
	Agree	31	
	Neutral	0	
	Disagree	0	
	Strongly disagree	0	
	**Rating scale 1–10**	**Response (%)**	
On a scale of 1 to 10, how would rate your learning experience after the course?	10/10	31	
	9/10	24.1	
	9/10	24.1	

For the question of what else could have been included in the course within the available time to enhance the learning experience of the students and what could have been done differently, omitted, or modified, mixed responses were received. Thematic analysis of the responses was done and the representative comments from the faculty and residents and the core ideas emerging from those comments are presented in Table 2 .

**Table 2:** Core ideas and representative comments of feedback received from faculty and residents

Source	Open-ended questions	Core ideas	Representative comments
Faculty	What else could have been included to enhance the learning experience of the students within the time available?	• More discussion • One topic in one course • Spotters, osteology sections • Inclusion of experts	“Format is quite similar to PG exams” “Active participation of other attending students” “More verbal exchange among all stakeholders—mentors, examiners, and residents” “Questions by some experts” “Total approach to the patients” “Time bound and relevant questions” “Update including recent advances” “Spotters, bones (osteology)” “Experts comment about the ideal management of the case” “Group discussion will make it better” “At one time, one topic could be covered exhaustively like, hand injuries alone, all types of clefts, or facial injuries along with various kinds of cases, so that one topic can be completely covered” “To increase the program's duration”
Residents		• Emphasis on planning part • More time for case discussion • Inclusion of videos—preoperative, flap planning • Specimen, more radiographs	“Exam situations can be better simulated virtually” “More emphasis on management part” “I should have taken better images and videos” “Standard pattern of each type of case could be handed over to the presenter” “Slot division—10 min for presentation and 20 min for discussion” “Increased duration of course, each case could be discussed for 1 h” “Being nicely done” “Short intraoperative video of the case” “Some flow charts” “Recording of patient's examination and flap planning” “Videos” “Specimen discussion, more discussion on long case” “More no. of radiographs in radiology section”
**Source**	**Open-ended questions**	**Core ideas**	**Representative comments**
Faculty	List three things that you did not like and could have been done differently, omitted, or modified, from your point of view	• Level of questions—both basic and advanced • Students' feedback of examiners • Time management for all aspects of the case • More interactive	“First- and second-year residents to get representation for presenting cases” “Everything should be time specific” “Students should also be given a feedback form (might be a different one) and asked for comments regarding the examiners” “Students should be given a certificate that they participated in the APSI mock examination and their performance was good/excellent; it will boost the confidence of the students” “MCh-specific Q/A should be taken care of” “Specific questions and appropriate answers should be taken care of, as basic questions to pass the candidate and relevant innovative ideas and recent advances after that for honors” “More questions from residents in the chatbox should be allowed” “Too less time spent on patient management planning” “Live demonstration of the patient” “Some faculty were interrupting the speaker too much, and too much time spent on inspection, and palpation when it may be more beneficial to spend time on essentials of management” “Group discussion in an operative session may be a better option”
Residents		• Omission of registration charges for nonmembers • Avoidance of technical glitches • Advance announcements • Lectures on difficult topics	“APSI nonmember resident registration charges could be omitted” “Delaying discussion more in the history part “More time to discuss” “Paying for PG to be omitted” “Reconsider the cost” “Lectures/seminars on difficult topics by speakers” “Liked most of them” “Announcements/alerts of the program in advance to read the topics” “Attendees could not be seen on Zoom” “Technical glitches by the presenting PG” “Long case too lengthy” “Cases selected to be standardized” “Network connectivity at my end”

**Fig. 3:**
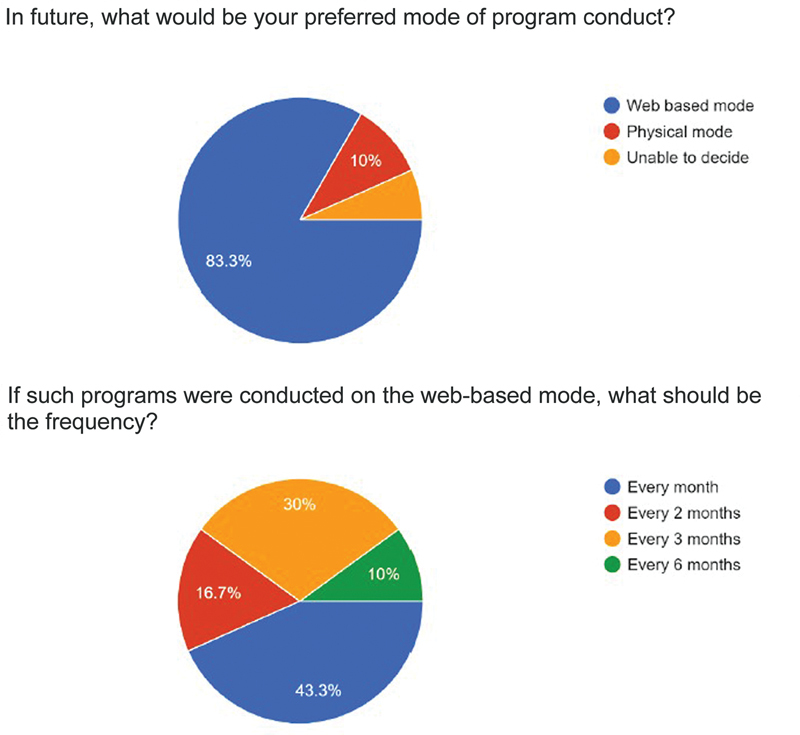
Distribution of responses for mode of conduct and frequency of APSI-PGME courses.

In response to the preferred mode of program conduct, 83.3% preferred it online, 10% preferred the physical mode, and 6.7% were unable to decide. Of those who preferred the online mode, 43.3% wanted the course every month, 16.7% every 2 months, 30% every 3 months, and 10% every 6 months ( Fig. 3 ).

## Discussion

Plastic surgery training has been challenging for a long time, teaching complex anatomy and pathophysiology, developing technical competence, and nurturing effective decision-making in a limited number of years. Although the operating room has long been regarded as an important location for learning and evaluating various competencies, classroom teaching, including resident case presentations, has high educational value but is often overlooked at a few centers. 6 So, to enhance the residents' confidence during examinations, regular case presentations not only in front of their teachers but also other examiners from different parts of the country can be helpful. The plastic surgery teachers must inculcate a few attributes in their students such as critical thinking and problem-solving skills, communication skills, collaboration, and creativity. 7 Creativity and innovation are an integral part of plastic surgery training; without these, the field could be replaced by artificial intelligence or robots. Collaboration is essential in plastic surgery because teamwork and knowledge exchange work like oxygen for the specialty. Communication skills not only in terms of expression during case presentation but also in terms of how well they listen to the patient and their teachers/examiners and how well they converse in diverse environments and by using multiple media and technologies are essential. Critical thinking is a systematic problem-solving skill that requires increased concentration, profound analytical abilities, and improved thought processing. Both teaching and assessment strategies, well conducted and well supervised, can inculcate these four Cs (Creativity and innovation, Collaboration, Communication skills, and Critical thinking) that are vital for a future plastic surgeon. With this thought and vision, the APSI-PGME course was started to bring residents and faculty educational interactions under a bigger umbrella and provide an excellent platform to further enhance the learning experiences of the participants.

## Why Is APSI-PGME Course a Competency-Based Learning?

The module has been prepared in congruence with the objectives of the plastic surgery curriculum designed by the National Medical Commission, which covers all the three domains of learning: cognitive, affective, and psychomotor. 2 A competency-based education also requires assessment and feedback (from the participants) as its integral components. A formative assessment in the form of feedback was given by the examiners and the adequacy of the course was envisaged by the feedback responses received from the participants at the end of every course. According to 89.7% of the participants, the time allotted for each section was just right. In response to the question on whether the objective of the program to enhance knowledge of the participants was achieved, 55.2% strongly agreed and 41.4% agreed. The item on whether such a program will benefit the students in gaining confidence was strongly agreed by 69% and agreed by 31%. The highest rating of 10 was given by 31% of the students. This course would also help in standardizing the teaching and learning of trainees and practicing plastic surgeons.

## Online versus Offline Course

During the COVID-19 pandemic, we witnessed an exponential rise in web-based teaching and training, and even after the pandemic, classroom and bedside teaching, face-to-face workshops, and multidisciplinary discussion meetings are continued on virtual platforms as an adjunct to classroom teachings. The intriguing question is how effective online learning is as compared with classroom teaching. There is no clear consensus as the literature is divided on this issue, and few systematic reviews and meta-analyses are available that report that there is no evidence that classroom learning works better. Blended learning is one method that incorporates the advantages of both and is an optimum method for knowledge acquisition. 8 The majority of the faculty members and residents attending the APSI-PGME courses preferred the online mode of program conduct (83.3%).Only 10% opted for the physical mode and 6.7% were unable to decide. In response to the preferred frequency, 43.3% wanted the course every month, 16.7% every 2 months, 30% every 3 months, and 10% every 6 months. The literature is full of speculations that hybrid events will be the future norm with the cost-effectiveness of virtual meetings, a larger audience, and ease of joining from any place being the greatest advantages. 9 The addition of technology like augmented reality and integrated voice assistants will be a valuable feature of these platforms to make it more productive and interesting. 10

## Modifications and Future Planning

A value-added advantage of APSI-PGME course is the archiving and its free access to APSI members. Over a while since the first course started, several modifications have been made like the inclusion of preoperative and flap planning videos, strict adherence to time, experts' opinions, and comments regarding particular case management. These modifications have been made based on the suggestions received in response to the question on what else could have been included in the course within the available time to enhance the learning experience of the students and what could have been done differently, omitted, or modified. In future, we plan to include objective scoring, peer assessment, and self-corrective strategies to further enhance the participant's competence.

## Conclusion

The APSI-PGME course for plastic surgery residents is a structured program designed to cover topics pertinent to the specialty curriculum and is conducted on the pattern of exit examination preparations. The expected outcome of gain in the residents' confidence as a result of their knowledge enhancement in one-to-one discussions with faculty and mentors has been well achieved. In an era of competency-based medical education that focuses on competencies rather than the completion of the curriculum and provides an effective outcome-based strategy, the ASPI-PGME course is a bold initiative by the APSI. As a competency-driven process, it will benefit the residents and young plastic surgeons to become lifelong learners, which in turn will benefit our specialty and hence the whole society.
