# Supplementary material for: Association of Plastic Surgeons of India Postgraduate Medical Education (APSI-PGME) Course: How Far Have We Reached?
Source: Indian J Plast Surg. 2025 Feb 24;58(5):363–70. doi: 10.1055/s-0045-1804532 (PMC12578547; doi:10.1055/s-0045-1804532)
Supplement: Supplementary file 1 — Supplementary Material 1 [file 10-1055-s-0045-1804532-s2483010-1.pdf]

ASSOCIATION OF PLASTIC SURGEONS OF INDIA (APSI)

Post Graduate Medical Education (PGME) Program

APSI-PGME 2022

Prepared by:

Dr Veena Singh
Additional Professor & HOD
Burns & Plastic Surgery
All India Institute of Medical Sciences
Patna, Bihar
Member, APSI Executive Committee

Dr Ankur Bhatnagar
Additional Professor
Burns & Plastic Surgery
Sanjay Gandhi Postgraduate Institute of Medical Sciences
Lucknow, U.P.
Member, APSI Executive Committee

TABLE OF CONTENTS

S.No.	Topics	Page No.
1	Introduction of the concept	2-3
2	Learning Objectives	4
3	Overview of APSI-PGME program	5
4	Flow of program	6-7
5	Roles & Responsibilities<br>a) Postgraduate student<br>b) Faculty Examiners<br>c) Mentors/Observers	8-9
6	Benefits of the program<br>a) Postgraduates<br>b) Faculty<br>c) APSI	10
7	Financial Implications	11
8	List of Cases	12-13
9	Google Form for Registration	14
10	List of Centres/Examiners	15-24
11	References	25

Disclaimer: *The thought and inspiration for the above course has been derived from Regional Refresher Course (RRC) of the Association of Surgeons of India (ASI) which is being held from July-September every year as generally final exams start from Nov-Dec up to April.*

1. Introduction of the concept

Adult learning theory is rooted in andragogy which is the practice of teaching adults, as opposed to pedagogy, the practice of teaching children. The seven principles of adult learning include self-direction, transformation, experience, mentorship, mental orientation, motivation, and readiness to learn and is Learner oriented as compared to Teacher oriented in pedagogy learning.

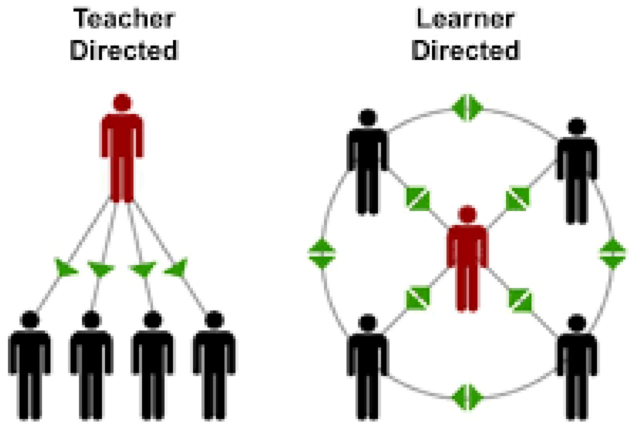

To foster the adult teaching learning skills in its postgraduate residents (MCh/DNB), Association of Plastic Surgeons of India (APSI) should have an ongoing teaching program round the year including the components of Self-Directed Learning which in turn, requires a number of skills, viz. critical thinking (problem solving) skills, research, effective time management, good ccommunication skills.

This Post Graduate Medical Education (PGME) for plastic surgery residents will be a structured program including case-based discussions (CBD), instruments, radiology, pathological specimens, operative viva. It will be designed in such an order to cover almost

all the topics pertinent to plastic surgery curriculum and conducted on the pattern of preparations for the exit exams. Apart from the responsibilities of teaching departments running these M.Ch./DNB programs, our esteemed association have taken upon itself to strengthen the academics of those PGs who are working at places where proper teaching and training is lacking. It will create an opportunity for the residents to appear for a mock viva-voice on a national platform in front of Faculty examiners from various centres and instil a feeling of confidence and healthy competition among the residents.

This will be entirely different from PG students presentations during Conferences and CMEs where they present their research work. Here they will be presenting like students and not researchers.

In the era of National Medical Commission (NMC) - Competency based Medical Education, which provides an effective outcome-based strategy and focused on competencies rather than the completion of curriculum, **PGME program would be novel initiative from APSI**. It will benefit the plastic surgery post graduate residents and young plastic surgeons so that they become life-long learners, which in turn will benefit our speciality and hence the whole society.

2. Learning Objectives

At the end of the each PGME program, the residents will be able to:

1. Recall the relevant anatomy and details of disease process.
2. Describe the clinical presentation of the patient.
3. Demonstrate the examination of the relevant part.
4. Describe the tests that are commonly done in clinical practice relevant to the diagnosis.
5. Understand the planning of particular case management.
6. Understand the type of surgical procedures in context to the patient presentation.
7. Demonstrate the decision-making skills in each case.
8. Write a short summary of the case.

3. Overview of APSI-PGME program

- The resident participating as Presenter in this program **MUST be APSI associate member.**
- The presenter may belong to any year of the MCh/DNB residency program.
- An online registration via Google form will be mandatory for participation.
- APSI will make all efforts to conduct these programs as Onsite meetings but in case of any issue, it will be held as web-based meeting.
- The deliberations **must not include any didactic lecture** and go beyond the clinical examination and include recent trends in the management of all cases to make it educational and informative to other plastic surgeons.
- These programs will also include **hands-on training in basic plastic surgical skills** e.g. suturing/microvascular/tendon/nerve repair and cadaveric flap dissections.
- Registration for all hands-on training courses should be separate and reserved only for the postgraduates. The other sessions including discussions can be attended by qualified plastic surgeons with prior registration.
- Feedback will be collected from all participants at the end of every such program so that further modifications may be done for maximal benefit.
- It can be conducted throughout the year on a zonal basis (3-4 in no.) so that if someone from one zone misses can join in the next zone.
- In future, APSI may also start the Best Resident Award in each Zone for a positive enforcement.

4. **Flow of program**

Each session will be conducted in four steps:

1. Presentation by PG student on a power point.
2. Questions will be asked by 2-3 Faculty Examiners.
3. Validation of the answers by Mentors, if required.
4. PG student may clarify the doubts at the end of the discussion.

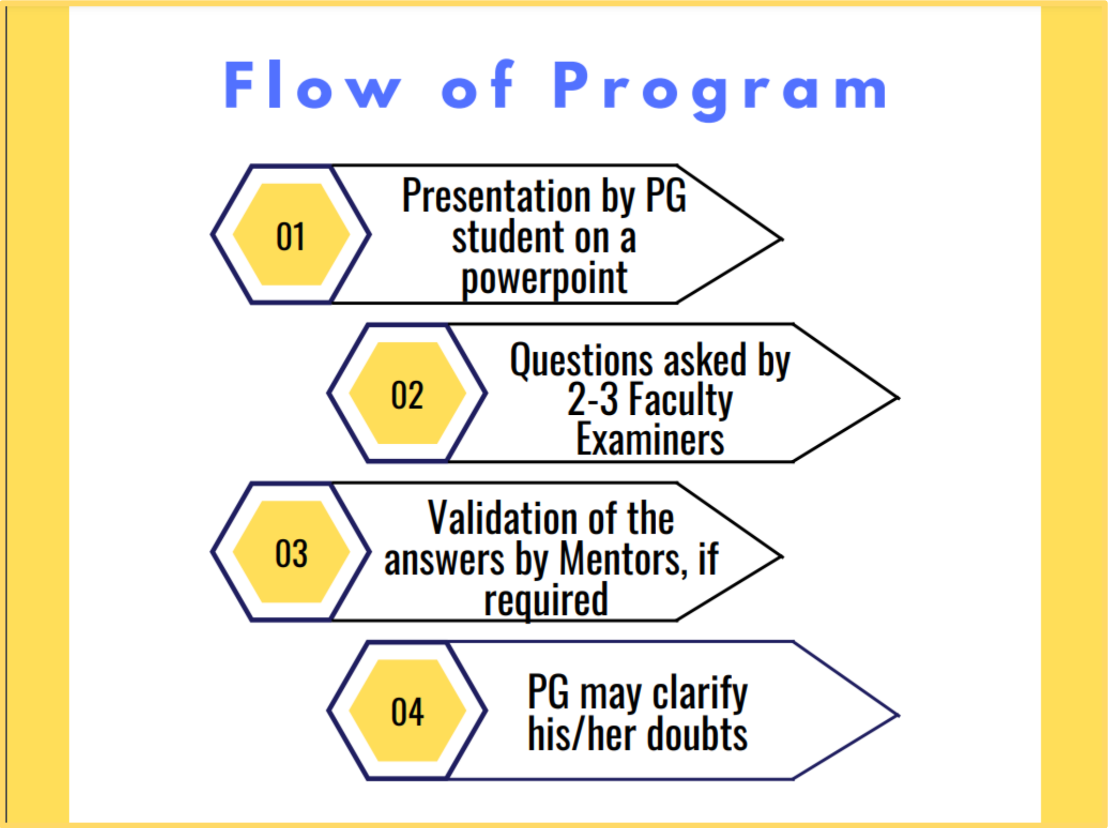

The flow of the complete program will remain same both in web-based and online mode. Modifications may be done from time to time depending upon the requirements and feedback.

Web-based meeting

Day 1: Long and short cases

Day 2: Radiology: CT/X- rays

Surgical instruments and devices

Pathology specimens/3D Models

Surgical procedures (operative viva)

Spotters

Onsite meeting

Day 1: Hands-on/Cadaveric workshop + Long and short cases

Day 2: Long and short cases

Radiology: CT/X- rays

Surgical instruments and devices

Pathology specimens/3D Models

Surgical procedures (operative viva)

Spotters

5. Roles and Responsibilities

a) Postgraduate students:

- The presenter student will present the long/short cases as a **powerpoint presentation in the same format as in classes/exams.**
- The student must be well read regarding the theoretical and practical aspects of the case.
- He/She should stick to the allotted time for presentation so that adequate time is there for discussion.

b) Faculty Examiners:

- Once the student has presented the case, a **panel of Faculty Examiners (2-3 in no.)** will cross-question the student.
- In case of incorrect answers by the student, the faculty will guide them towards correct concept and decision making.
- It will be the responsibility of the Faculty to complete the discussion within the allotted time.

c) Mentors/Observers:

- Mentor/observers will be the **senior examiners like Past Presidents of APSI** who will validate the answers in case there is no consensus among the Faculty Examiners.
- The presence of a few senior examiners should be an integral part as they are the ones who can standardize the answers so that students from any center will come to know the standard answers to every question rather than getting confused.

6. Benefits of the program

a) Benefits to the PGs:

- It will give them a boost for preparing well for their practical/viva voice (like SDL, Self-Directed Learning).
- It will create a competitive environment among the PG students from various centres.
- It will help them in providing validated answers (validation will be done by Faculty Examiners and Mentors who have been regular examiners).
- It will bring uniformity in their knowledge, gap and practices (irrespective of their training centre).

b) Benefits to the Faculty Examiners/Mentors:

- Opportunity to teach other students apart from their own centres.
- Feeling of satisfaction on seeing the students more empowered.
- The biggest benefit will be to pursue the passion of a teacher.

Learning is best when done in collaboration

7. Financial Implications

- Approximate expenses for **one ONLINE session** (as per market survey from few vendors) is:

  INR 8,000 – 12,000/Session (including GST)

- One session will consist of 2 hours/day for 2 consecutive days.
- 3 – 4 such sessions may be planned in a year. Increase in the no. of sessions will depend upon the feedback from participants and Faculty.
- The financial implications of sessions held as physical meetings will be borne by the Organizers.

8. List of Items (*)

A. Long Cases

1. Lowe limb defect with exposed bone
2. Post burn contracture neck
3. Carcinoma oral cavity
4. Pressure sore
5. Brachial plexus injury

B. **Short Cases**

1. Cleft lip/palate
2. Facial nerve palsy
3. Hypospadias
4. Gynaecomastia
5. Ulnar nerve palsy
6. Median nerve palsy
7. Radial nerve palsy
8. Post burn contracture hand
9. Post burn contracture knee
10. Post burn contracture Axilla
11. Vascular malformation
12. Ptosis
13. Syndactyly

14. Basal cell carcinoma
15. Squamous cell carcinoma
16. Flexor tendon injury
17. Extensor tendon injury
18. Dupuytren’s contracture
19. Camptodactyly
20. Constriction ring syndrome
21. Microtia
22. Cut injury nose/Cleft nose
23. TMJ Ankylosis

*** Any other cases at the discretion of course directors to the existing list**

C. Radiology

D. X-rays. OPD, CT, MRI

E. Instruments + Operative viva

F. Specimen/3D Models

G. Spotters

9. Google Form for Registration

9/20/22, 9:21 PM Association of Plastic Surgeons of India APSI-PGME Registration Form 2022

Association of Plastic Surgeons of India APSI-PGME Registration Form 2022

Date: 13-14th October 2022
Timing: 8 PM - 10 PM (both days)

* Required

1. **Name** *

2. **Mobile no** *

3. **Email** *

4. **College/Institute** *

5. **State**

6. **Faculty** *

Mark only one oval.

- APSI Member
- Non APSI Member

https://docs.google.com/forms/d/1jBjG0C5DGXcl5PuXCNZfBalbtlH4J5p7lXlcLAHYNe8/edit 1/2

9/20/22, 9:21 PM Association of Plastic Surgeons of India APSI-PGME Registration Form 2022

7. **PG Student** *

Mark only one oval.

- APSI Member
- Non APSI Member
- Applied for membership

Please feel free to contact on:
drsveena@aiimspatna.org
bhatnagarankur2000@yahoo.com

This content is neither created nor endorsed by Google.

Google Forms

https://docs.google.com/forms/d/1jBjG0C5DGXcl5PuXCNZfBalbtlH4J5p7lXlcLAHYNe8/edit 2/2

10. List of MCh/DNB Centres (Ref: APSI Website)

(Please visit www.nmc.org.in for updated information)

Post Graduate Courses (Plastic Surgery) in India MCh

Sr. No.	Course Name	State	Name and Address of Medical College / Medical Institution	Annual Intake (Seats)
1	M.Ch - Plastic Surgery	Andhra Pradesh	Andhra Medical College, Visakhapatnam	2
2	M.Ch - Plastic Surgery	Andhra Pradesh	Narayana Medical College, Nellore	2
3	M.Ch - Plastic Surgery	Assam	Gauhati Medical College, Guwahati	2
4	M.Ch - Plastic Surgery	Bihar	Patna Medical College, Patna	1
5	M.Ch - Plastic Surgery	Chandigarh	Postgraduate Institute of Medical Education & Research, Chandigarh	10
6	M.Ch - Plastic Surgery	Delhi	PGIMER Dr. RML Hospital, New Delhi	4

7	M.Ch - Plastic Surgery	Delhi	Vardhman Mahavir Medical College & Safdarjung Hospital, Delhi	10
8	M.Ch - Plastic & Reconstructive Surgery	Delhi	All India Institute of Medical Sciences, New Delhi	15
9	M.Ch - Plastic Surgery	Gujarat	B J Medical College, Ahmedabad	4
10	M.Ch - Plastic Surgery	Gujarat	Government Medical College, Surat	1
11	M.Ch - Plastic Surgery	Gujarat	Medical College, Baroda	2
12	M.Ch - Plastic Surgery	Gujarat	MP Shah Medical College,Jamnagar	1
13	M.Ch - Plastic Surgery	Gujarat	SBKS Medical Instt. & Research Centre, Vadodra	1
14	M.Ch - Plastic Surgery	Gujarat	Smt. N.H.L.Municipal Medical College, Ahmedabad	8
15	M.Ch - Plastic Surgery	Haryana	Pt. B D Sharma Postgraduate Institute of Medical Sciences, Rohtak (Haryana)	1

16	M.Ch - Plastic Surgery	Jammu & Kashmir	Sher-I-Kashmir Instt. Of Medical Sciences, Srinagar	2
17	M.Ch - Plastic Surgery	Karnataka	A J Institute of Medical Sciences & Research Centre, Mangalore	1
18	M.Ch - Plastic Surgery	Karnataka	Bangalore Medical College and Research Institute, Bangalore	4
19	M.Ch - Plastic Surgery	Karnataka	Jawaharlal Nehru Medical College, Belgaum	1
20	M.Ch - Plastic Surgery	Karnataka	M S Ramaiah Medical College, Bangalore	1
21	M.Ch - Plastic Surgery	Karnataka	Rajarajeswari Medical College & Hospital, Bangalore	1
22	M.Ch - Plastic Surgery	Karnataka	S S Institute of Medical Sciences& Research Centre, Davangere	1
23	M.Ch - Plastic Surgery	Karnataka	St. Johns Medical College, Bangalore	2
24	M.Ch - Plastic Surgery	Karnataka	Vydehi Institute Of Medical Sciences & Research Centre, Bangalore	2
25	M.Ch - Plastic Surgery	Kerala	Amrita School of Medicine, Elamkara, Kochi	2

26	M.Ch - Plastic Surgery	Kerala	Government Medical College, Kottayam	1
27	M.Ch - Plastic Surgery	Kerala	Government Medical College, Kozhikode, Calicut	2
28	M.Ch - Plastic Surgery	Kerala	Medical College, Thiruvananthapuram	2
29	M.Ch - Plastic Surgery	Maharashtra	Armed Forces Medical College, Pune	3
30	M.Ch - Plastic Surgery	Maharashtra	B. J. Govt. Medical College, Pune	1
31	M.Ch - Plastic Surgery	Maharashtra	Government Medical College, Nagpur	0
32	M.Ch - Plastic Surgery	Maharashtra	Grant Medical College, Mumbai	4
33	M.Ch - Plastic Surgery	Maharashtra	Krishna Institute of Medical Sciences, Karad	1
34	M.Ch - Plastic Surgery	Maharashtra	Lokmanya Tilak Municipal Medical College, Sion, Mumbai	2
35	M.Ch - Plastic Surgery	Maharashtra	Mahatma Gandhi Missions Medical College, Aurangabad	1

36	M.Ch - Plastic Surgery	Maharashtra	N. K. P. Salve Instt. of Medical Sciences and Research Centre and Lata Mangeshkar Hospital, Nagpur	1
37	M.Ch - Plastic Surgery	Maharashtra	Padmashree Dr. D.Y.Patil Medical College, Navi Mumbai	1
38	M.Ch - Plastic Surgery	Maharashtra	Seth GS Medical College, Mumbai	2
39	M.Ch - Plastic Surgery	Maharashtra	Tata Memorial centre, Mumbai	2
40	M.Ch - Plastic Surgery	Maharashtra	Topiwala National Medical College, Mumbai	4
41	M.Ch - Plastic & Reconstructive Surgery	Manipur	Regional Institute of Medical Sciences, Imphal	1
42	M.Ch - Plastic Surgery	Orissa	SCB Medical College, Cuttack	3
43	M.Ch - Plastic Surgery	Pondicherry	Jawaharlal Institute of Postgraduate Medical Education & Research, Puducherry	2
44	M.Ch - Plastic Surgery	Pondicherry	Pondicherry Institute of Medical Sciences & Research, Pondicherry	1

45	M.Ch - Plastic Surgery	Punjab	Christian Medical College, Ludhiana	2
46	M.Ch - Plastic Surgery	Punjab	Dayanand Medical College & Hospital, Ludhiana	2
47	M.Ch - Plastic Surgery	Rajasthan	SMS Medical College, Jaipur	6
48	M.Ch - Plastic & Reconstructive Surgery	Rajasthan	Mahatma Gandhi Medical College and Hospital, Jaipur	1
49	M.Ch - Plastic Surgery	Tamil Nadu	Chengalpattu Medical College, Chengalpattu	1
50	M.Ch - Plastic Surgery	Tamil Nadu	Christian Medical College, Vellore	3
51	M.Ch - Plastic Surgery	Tamil Nadu	Coimbatore Medical College, Coimbatore	2
52	M.Ch - Plastic Surgery	Tamil Nadu	Kilpauk Medical College, Chennai	6
53	M.Ch - Plastic Surgery	Tamil Nadu	Madras Medical College, Chennai	8
54	M.Ch - Plastic Surgery	Tamil Nadu	Madurai Medical College, Madurai	2

55	M.Ch - Plastic Surgery	Tamil Nadu	Sri Ramachandra Medical College & Research Institute, Chennai	2
56	M.Ch - Plastic Surgery	Tamil Nadu	SRM Medical College Hospital & Research Centre, Kancheepuram	1
57	M.Ch - Plastic Surgery	Tamil Nadu	Stanley Medical College, Chennai	6
58	M.Ch - Plastic Surgery	Tamil Nadu	Thanjavur Medical College,Thanjavur	1
59	M.Ch - Plastic Surgery	Telangana	Deccan College of Medical Sciences, Hyderabad	1
60	M.Ch - Plastic Surgery	Telangana	Gandhi Medical College, Secunderabad	3
61	M.Ch - Plastic Surgery	Telangana	Nizams Institute of Medical Sciences, Hyderabad	4
62	M.Ch - Plastic Surgery	Telangana	Osmania Medical College, Hyderabad	8
63	M.Ch - Plastic Surgery	Uttar Pradesh	Institute of Medical Sciences, BHU, Varansi	2
64	M.Ch - Plastic Surgery	Uttar Pradesh	Jawaharlal Nehru Medical College, Aligarh	4

65	M.Ch - Plastic Surgery	Uttar Pradesh	King George Medical University, Lucknow	4
66	M.Ch - Plastic Surgery	West Bengal	Institute of Postgraduate Medical Education & Research, Kolkata	6
67	M.Ch - Plastic Surgery	West Bengal	Medical College, Kolkata	4
68	M.Ch - Plastic Surgery	West Bengal	Nilratan Sircar Medical College, Kolkata	1
69	M.Ch - Plastic Surgery	West Bengal	RG Kar Medical College, Kolkata	3

Post Graduate Courses (DNB Plastic Surgery) in India:

Sr. No.	Name of the Hospital	Address of the institution	Annual Intake (Seats)
1	Army Hospital (R & R)	Near Dhaulakuan Metro Station, Delhi Cantt - 110010	2
2	Lok Nayak Jayprakash Narayan Hospital	NEW DELHI-110002	2

3	Maharaja Agarsen Hospital	Punjabi Bagh (General Store) NEW DELHI-26	1
4	Sir Ganga Ram Hospital	Rajinder Nagar NEW DELHI-60	1
5	Manipal Hospital	98 Rustum Bagh Airport Road BANGALORE - 17	1
6	Sparsh Hospital	29/P2 The Health City Hosur Road Bommasandra Bangalore-560099	1
7	Bangalore Medical College & Research Institue	Fort Bangalore - 560002	4
8	Amrita Institute of Medical Scs. Amrita	Elamkara (PO) KOCHI-26 Kerala	1
9	Medical Trust Hospital	M. G. Road KOCHI-16 Kerala	1
10	Specialists Hospital	Kochi	0
11	Elite Mission Hospital	Koorkkan cherry Thrissur - 7	1
12	Bombay Hospital & Instt. of Med. Science	12 Marine Lines MUMBAI-20	2
13	Deenanath Mangeshkar Hospital & Res. Cen	Erandwane PUNE - 4	1

14	Amandeep Hospital	Amandeep Hospital G.T.Road Model Town Amritsar	1
15	Apollo First Med Hospital	No.154 Poonamallee High Road Kilpauk Chennai-600010	2
16	Right Hospital	1 Professor Subramaniam Street Kilpauk Chennai-10	1
17	Ganga Medical Centre	Ganga Medical Centre and Hospital 313, Mettupalayam Road, Coimbatore - 641043	3
18	Vivekananda Polyclinic	Vivekanandpuri LUCKNOW - 7	1

11. References:

1. https://asiindia.org/2021/05/28/regional-refresher-courses-rrc-2021
2. David C. M. Taylor & Hossam Hamdy (2013) Adult learning theories: Implications for learning and teaching in medical education: AMEE Guide No. 83, Medical Teacher, 35:11, e1561-e1572, DOI: 10.3109/0142159X.2013.828153.
3. https://apsi.in/post-graduate
4. https://www.nmc.org.in/information-desk/college-and-course-search
