# Supplementary material for: Association of Plastic Surgeons of India Postgraduate Medical Education (APSI-PGME) Course: How Far Have We Reached?
Source: Indian J Plast Surg. 2025 Feb 24;58(5):363–70. doi: 10.1055/s-0045-1804532 (PMC12578547; doi:10.1055/s-0045-1804532)
Supplement: Supplementary file 2 — Supplementary Material 2 [file 10-1055-s-0045-1804532-s2483010-2.pdf]

List of cases discussed in the APSI-PGME Courses till date					
	**Long cases**	**Short cases**	**Instruments**	**Radiology**	**Operative viva**
Course I<br>13-14th October 2022	Ca oral cavity	Radial nerve palsy<br>PBC hand<br>Syndactyly<br>Hypospadias	Skin grafting set<br><br>Cleft palate set	OPG	Harvest of ALT flap
Course II<br>28-29th December 2022	Lower leg defect	Cleft palate<br>Ulnar nerve palsy<br>Microtia<br>Cleft lip<br>BCC upper lip	Microsurgery set	Polydactyly/syndactyly	Free radial artery flap
Course III<br>20th -21st April 2023	PBC Neck	Gynecomastia<br>TMJ ankylosis<br>Ischial pressure sore<br>Macrodactyly<br>SCC lower limb	Facial fracture instruments set	Facial bones	Millard's lip repair
Course IV<br>27-28th July 2023	Brachial plexus injury	Ear defect<br>Nasal defect<br>Facial nerve palsy<br>Microtia<br>Radial nerve palsy	Skin grafting set	Polydactyly/radial longitudinal defect	Modified Mohler cleft lip repair
Course V<br>28-29th September 2023	Lower leg defect	SCC scalp<br>Ca lower lip<br>Ulnar nerve palsy<br>Facial cleft<br>Facial nerve palsy	Cleft palate set	Hand fractures	Harvest of free fibula
Course VI<br>30th November - 1st December 2023	PBC Neck	Radial nerve palsy<br>Microtia<br>BCC face<br>Flexor tendon injury<br>Scalp defect	Rhinoplasty set	CT face	Bardach's palatoplasty
Course VII<br>14-15th March 2024	Ca oral cavity	PBC axilla<br>Nasal ala defect<br>PB face and ectropion eyelid<br>Marjolin's ulcer foot<br>Cleft palate	Microsurgery set	Polydactyly/syndactyly	Tendon transfer for ulnar nerve palsy
Course VIII<br>27-28th June 2024	Brachial plexus injury	Macrostomia<br>Nasal defect<br>Radial nerve palsy<br>Flexor tendon injury<br>SCC lower leg	Cleft instruments set	X-ray hand	Asopa II
