# Supplementary material for: Association of Plastic Surgeons of India Postgraduate Medical Education (APSI-PGME) Course: How Far Have We Reached?
Source: Indian J Plast Surg. 2025 Feb 24;58(5):363–70. doi: 10.1055/s-0045-1804532 (PMC12578547; doi:10.1055/s-0045-1804532)
Supplement: Supplementary file 3 — Supplementary Material 3 [file 10-1055-s-0045-1804532-s2483010-3.pdf]

Feedback: APSI-PGME Course

Dear Friends,

Greetings!
We hope that all of you had a fruitful and enjoyable learning experience in the APSI-PGME Course. Please complete this questionnaire, to provide critical, honest and constructive feedback which would be valuable to improve future sessions. Completing this online survey would be considered as implied consent for using the response for educational purposes.

Warm regards,
Course Directors, APSI-PGME Course

* Indicates required question

1. **You participated in the program as** *

Mark only one oval.

- Presenter PG
- Attending PG
- Faculty Teacher/ Examiner
- Mentor/Observer
- Consultant

2. **Year of Residency (For PGs Only)**

____

3. **Are you APSI member** *

Mark only one oval.

- Yes
- No
- Have Applied

Section 2

4. Time alloted for each section was *

Mark only one oval.

- Too fast
- Fast
- Just right
- Slow
- Too slow

5. The objective of the program to enhance the knowledge of participants was achieved? *

Mark only one oval.

- Strongly agree
- Agree
- Neutral
- Disagree
- Strongly disagree

6. Such program will benefit the students in gaining confidence *

Mark only one oval.

- Strongly agree
- Agree
- Neutral
- Disagree
- Strongly Disagree

7. On a scale n 1 to 10, how would you rate the standard of the progam? *

Mark only one oval.

	1	2	3	4	5	6	7	8	9	10	
Leas	◯	◯	◯	◯	◯	◯	◯	◯	◯	◯	Most useful

Section 3

8. Within the time available, what else could have been included to enhance the learning experience of the students *

____
____
____
____
____

9. List three things which you did not like and could have been done differently, omitted or modified, from your point of view *

____
____
____
____
____

10. In future what would be your preferred mode of program conduct *

Mark only one oval.

- ◯ Web based mode
- ◯ Physical mode
- ◯ Unable to decide

11. If such program would conduct on web based mode what should be the frequency *

Mark only one oval.

- Every month
- Every 2 months
- Every 3 months
- Every 6 months

This content is neither created nor endorsed by Google.

Google Forms
